# Omics Analysis Revealing Flavonoid Content During Maize Grain Germination

**DOI:** 10.3390/metabo15020107

**Published:** 2025-02-07

**Authors:** Xiaomin Tian, Lirong Chen, Linlin Sun, Kuijie Gong, Kaichang Liu, Yuqiu Guo

**Affiliations:** 1 Crop Research Institute, Shandong Academy of Agricultural Sciences, North Industrial Road 202, Jinan 250100, China; txm17862686919@163.com (X.T.); 13047481469@163.com (L.C.); linlins4355@163.com (L.S.); gongkj@gmail.com (K.G.); 2 Shandong Academy of Agricultural Sciences, North Industrial Road 202, Jinan 250100, China; liukc1971@163.com

**Keywords:** maize grain, transcriptome, metabolomics, differentially expressed genes, differentially expressed metabolites

## Abstract

**Background/Objectives:** The germination process initiates an active process of secondary metabolism, which produces a series of secondary metabolites, including flavonoids. **Methods:** A metabolomics and transcriptomics analysis was conducted on maize grains germinated at three different stages. **Results:** A total of 374 metabolites were detected in maize grains. From the raw maize grain to various stages of germination, 3 anthocyanins, 61 flavones, 12 flavonols, 13 flavanones, and 6 isoflavones were identified, respectively. An integrated omics analysis discovered that a total of 16 flavonoid metabolites were mapped to 4 KEGG pathways, which were associated with 40 related genes. This indicates that germination has significant benefits in improving the nutritional function of corn kernels. **Conclusions:** In summary, the findings of this study provide valuable insights into flavonoid metabolites and related genes, demonstrating the profound impact of germination treatment on the nutritional and functional aspects of maize grains.

## 1. Introduction

The application of germination technology has garnered increasing attention due to its ability to not only enhance the nutritional, functional, and bioactive components of grains but also improve their sensory properties. Consequently, germination emerges as a green food engineering method for accumulating natural bioactive compounds. Certain edible seeds and sprouts, rich in these compounds, can serve as functional foods for preventing chronic diseases [1,2]. Antioxidants, minerals, vitamins, and dietary fibers in grains are all influenced by germination [3], with edible sprouts being excellent sources of phenolic antioxidants [4]. Significant changes in bioactive compounds, vitamins, γ-aminobutyric acid, and polyphenols, coupled with enhanced antioxidant capacity, occur in edible seeds post-germination [5], making them valuable ingredients for food and pharmaceutical products.

Currently, nearly all grains undergo germination, including millet [6,7], brown rice [8], wheat [9], corn [10], legume seeds [11], sorghum [12], oats, barley, rye, and buckwheat [13]. For legumes and rice varieties, fatty acid profiles shift post-germination [14]. Substituting germinated wheat flour increases folate content in flour and bread by 1.5–4 times, depending on the substitution level [15]. During germination, phytase activity increases in rice, corn, millet, sorghum, and wheat [16], with some grains and legume seeds experiencing a 3–5-fold increase [17]. However, Nour et al. [18] found that germinated sorghum significantly reduced tannins and phytate while increasing digestible protein (*p* < 0.05). Other studies show that wheat grain germination enhances functional components like α-tocopherol, certain minerals, and fatty acids [19], and improves oat’s nutritional properties [20].

Germination markedly elevates tocopherols, niacin, riboflavin, and levels of free and bound phenolic compounds, thereby enhancing the nutritional value and antioxidant capacity of wheat grains/flour. As a rich source of bioavailable phytochemicals, germinated wheat finds applications in enhancing the nutritional quality of food [21].

Corn (*Zea mays* L.), the third most significant cereal crop globally after rice and wheat, is primarily utilized in poultry and animal husbandry. However, in numerous developing countries, it remains a vital source of food security. For corn kernels, germinating under optimal conditions is an effective strategy to enhance their nutritional quality and value [22]. This process leads to increased protein, lysine, tryptophan, and neutral detergent fiber content, while in vitro protein digestibility decreases with the number of days post-germination [23]. Germination results in augmented starch and lysine content, along with increased reducing sugars and insoluble dietary fiber, but decreased protein content and soluble dietary fiber [24]. It also alters the ratio of crystalline to amorphous regions in corn starch, disrupting its microstructure and reducing starch granule size. Furthermore, five protein bands in germinated corn kernels exhibit higher concentrations than those in ungerminated kernels [25]. Acid phosphatase activity increases during the first 24 h of corn seed germination [26]. Germination and ultra-fine grinding can significantly elevate gluten content and expansion properties of corn flour [27] (*p* < 0.05).

Flavonoids, phenolic low molecular weight plant secondary metabolites with high antioxidant capacity, are widely distributed throughout the plant kingdom [28]. Flavonoids serve multiple functions in plants, including UV and visible light protection, safeguarding cells and tissues from the detrimental effects of reactive oxygen species, pathogen defense, and interspecific communication [29,30]. Recent reports have also provided compelling evidence for the crucial role of flavonoids in growth and development [31] and their inhibitory effect on auxin transport [32].

For centuries, flavonoid compound preparations containing physiologically active ingredients have been utilized in the treatment of human diseases [33], such as inflammation, heart disease, cancer [34], among others. Nowadays, individuals strive to balance their daily diets through natural products, fostering healthy eating habits. Flavonoids continually attract attention due to their vital antioxidant properties.

The combined analysis of transcriptomics and metabolomics was identified as an effective approach to elucidating metabolism-related functional genes [35]. However, to date, there is a scarcity of literature on flavonoids in germinated corn seeds, and no studies have reported on the transcriptomic and metabolomic analysis of flavonoids in germinated corn kernels. Therefore, the aim of this study is to integrate metabolomics and transcriptomics to unravel the flavonoid germination process in corn kernels. As corn is a crucial food and fuel crop, and flavonoids are significant metabolites in corn kernels, a comprehensive study of their metabolome and transcriptome holds great significance for enhancing the functional properties of corn kernels.

## 2. Materials and Methods

### 2.1. Plant Materials and Treatment

The yellow corn (*Zea mays* L.) kernels were purchased locally in Jinan, Shandong. After sterilization with sodium hypochlorite (0.5%, *m*/*v*) for 0.5 h, 600 kernels were soaked in distilled water for 8 h. Following the removal of excess water, 200 kernels were frozen in liquid nitrogen and stored at −80 °C for future use. The remaining 400 kernels were germinated in a constant temperature and humidity incubator (160 HL, Jinyi Instrument, Changzhou, China) at 25 °C with 90% relative humidity. At the grainling stage and 12 h after germination, 200 kernels were frozen in liquid nitrogen and stored at −80 °C for later use.

### 2.2. Metabolite Extraction and LC-ESI-MS/MS System

The freeze-dried corn kernels were pulverized using a mixed mill (MM 400, Retsch, Shanghai, China) with zirconia beads at 30 Hz for 1.5 min. A 100 mg sample was extracted overnight with 70% methanol aqueous solution at 4 °C, followed by centrifugation at 10,000× *g* for 10 min. Before LC-MS analysis, the extract was absorbed (CNWBOND Carbon-GCB SPE Cartridge, 250 mg, 3 mL; ANPEL, Shanghai, China) and filtered (SCAA-104, 0.22 μm pore size; ANPEL, Shanghai, China). Quality control (QC) samples were prepared by mixing equal volumes of all samples, and one QC sample was injected every 10 injections during the measurement to monitor the stability of analytical conditions.

Sample extraction was performed using an LC-ESI-MS system (HPLC, Shimpack UFLC SHIMADZU CBM 30 A system; MS, Applied Biosystems 6500 Q TRAP, Waters, Milford, MA, USA). The HPLC column was C 18 (Waters ACQUITY UPLC HSS T 3, 1.8 μm, 2.1 mm × 100 mm, Waters, Milford, MA, USA), and the binary solvent system consisted of ultrapure water containing 0.04% acetic acid as mobile phase A and acetonitrile containing 0.04% acetic acid as mobile phase B. The A:B (*v*/*v*) gradient was set as 95:5 (0 min), 5:95 (11.0 min), 5:95 (12.0 min), 95:5 (12.5 min), and 95:5 (15.0 min). The flow rate was maintained at 0.40 mL/min, the column temperature at 40 °C, and the injection volume at 2 μL.

The ESI source operating parameters were set as follows: ion source ESI+, turbo spray; source temperature 500 °C; ion spray voltage (IS) 5500 V; ion source gas I (GSI), gas II (GSII), and curtain gas (CUR) were set to 55, 60, and 25 psi, respectively; and collision gas (CAD) was at high pressure. Instrument tuning and mass calibration were performed using 10 and 100 μmol/L polypropylene glycol solutions in QQQ and LIT modes, respectively. QQQ scans were acquired in MRM experiments with collision gas (nitrogen) at 5 psi.

### 2.3. Metabolite Identification and Quantification

Metabolite identification was based on the self-compiled database MWDB (Met Ware Biological Science and Technology Co., Ltd., Wuhan, China). During the identification process, repeated signals of large molecular weights such as K^+^, Na^+^, and NH^4+^ were eliminated.

Metabolite quantification was performed using the MRM (Multiple Reaction Monitoring) mode with a detection time of 80 s and a target scan time of 1.5 s. The quadrupole filtered the precursor ions (parent ions) of target substances, excluding ions corresponding to other molecular weights to prevent interference. After obtaining metabolite data from different samples, the mass spectrometry peak areas of all substances were integrated, and the mass spectrometry of the same metabolites in different samples was corrected.

Partial Least Squares-Discriminant Analysis (PLS-DA) was conducted on the identified metabolites, and a variable importance in projection (VIP) threshold of ≥1 and a fold change threshold of ≥2 or ≤0.5 were set for metabolites with significantly different contents.

### 2.4. RNA Extraction and Illumina Sequencing

Total RNA was extracted from corn kernel samples using the CTAB method [36]. The quantity and quality of RNA were determined by a Nano Drop ND 1000 spectrophotometer (Nano Drop Technologies, Wilmington, DE, USA) and an Agilent Bioanalyzer 2100 system (Agilent Technologies, Palo Alto, CA, USA), respectively. The integrity of RNA was assessed by 1% agarose gel electrophoresis, and the RNA concentration was adjusted to be uniform. mRNA was isolated from total RNA using magnetic beads with oligonucleotides (dT). cDNA was synthesized using a cDNA synthesis kit, and sequencing adapters were attached to both ends [37]. Library preparation and sequencing were performed on the Illumina HiSeqTM 2500 platform. Sequence data with base pair quality of Q ≥ 20 were extracted, and filtered reads were mapped to the maize B73 reference genome URL (8 July 2024). FPKM was used for quantification at the transcript level, and DEGs were identified using the edge R package (https://download.maizegdb.org/B73_RefGen_v3/ (accessed on 1 January 2025)) based on raw count data, with |log_2_ fold change| ≥ 1 and adjusted *p* ≤ 0.005.

### 2.5. Integrated Analysis of Metabolome and Transcriptome

Metabolites and DEGs involved in flavonoid biosynthesis in KEGG pathways were selected for comprehensive analysis. Metabolites for correlation analysis were screened based on VIP > 1, *p* < 0.05, and |log_2_ fold change| ≥ 1. The Pearson correlation coefficient and *p*-value for the integration of metabolome and transcriptome data were calculated using the Spearman method [5].

## 3. Results and Discussion

### 3.1. Analysis of Metabolites in Germinated Corn Kernels

**Figure A1:**
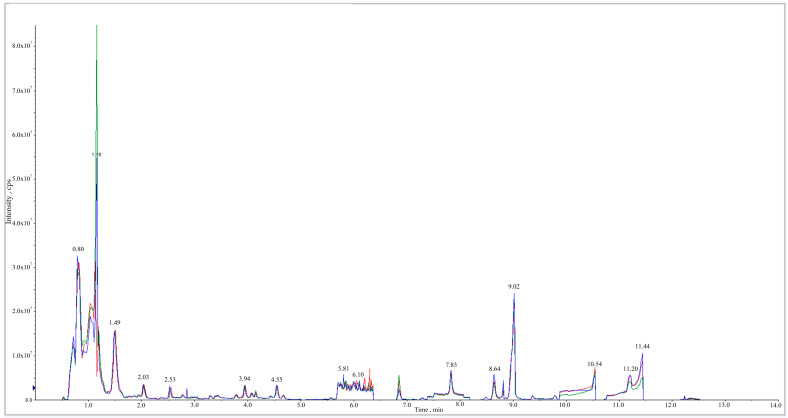
Total ion chromatograms of the metabolome of CK, GL, and YL corn kernels. Three lines of different colors indicate good repeatability of three samples.

To evaluate the effects of germination on the nutrition and functionality of corn kernels, the contents of metabolites at three different stages of germinated corn kernels were analyzed using UPLC-MS/MS. Under the MRM mode, the total ion current (TIC) curves of the three quality control (QC) samples showed high overlap (Appendix A Figure A1), indicating the reliability of the established method and good instrument stability. A total of 611 metabolites were detected in corn kernels.

**Figure 1:**
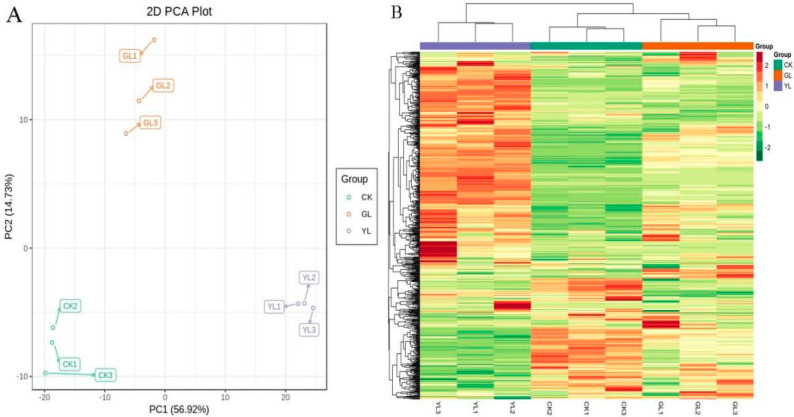
PCAof metabolome of CK, GL, and YL corn kernels (**A**). Heatmap analysis of metabolites in CK, GL, and YL corn kernels (**B**).

Principal Component Analysis (PCA) was employed to identify differences in metabolites among raw maize kernel, germination period, and sprouting period of corn kernels. Figure 1A presents the results of the three stages in a two-dimensional manner, where the first two principal components explain 56.92% and 14.73% of the variance between samples, respectively. PCA of the metabolome revealed distinct differentiation of metabolites in corn kernels at the three different stages. Figure 1B displays a heatmap of different metabolite data (three replicates per stage), which is consistent with the PCA results.

**Figure 2:**
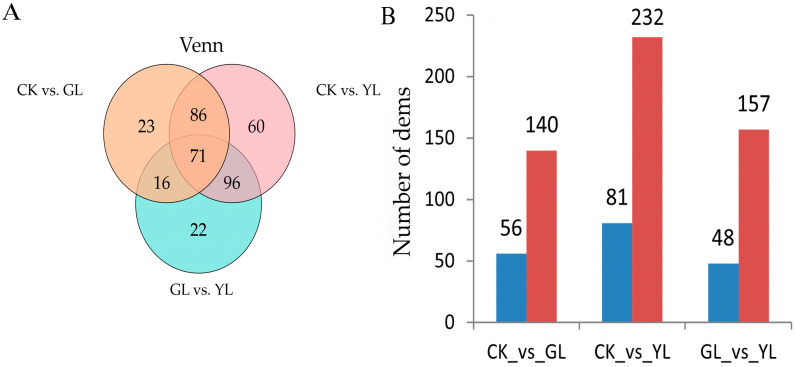
Venn diagram analysis of CK, GL, and YL (**A**). Number of dems in CK, GL, and YL (**B**).

Among the three groups of CK (raw maize kernel) vs. GL (germination period), CK vs. YL (sprouting period), and GL vs. YL, 196, 313, and 205 differentially expressed metabolites (dems), respectively, were detected. When comparing two different stages, 56, 81, and 48 metabolites were downregulated, while 140, 232, and 157 metabolites were upregulated in CK vs. GL, CK vs. YL, and GL vs. YL, respectively (Figure 2B). A Venn diagram analysis showed that there were 71 common dems among the 3 control groups (Figure 2A).

### 3.2. Analysis of Flavonoid Metabolite Categories

Flavonoids, a representative group of polyphenolic secondary metabolites, are widely distributed in the plant kingdom and have garnered significant attention over time. To date, over 8000 distinct flavonoid compounds have been identified [38]. Based on their fundamental molecular structures, flavonoids can be classified into six major groups: flavones, flavonols, flavanones, isoflavones, anthocyanins, and anthocyanidins [39]. This study identified a total of 95 flavonoid compounds across the CK, GL, and YL samples, each with 3 biological replicates, consisting of 3 anthocyanins, 61 flavones, 12 flavonols, 13 flavanones, and 6 isoflavones (Table 1). Applying the significant difference thresholds of VIP ≥ 1.0 and |log2 fold change| ≥ 2 or ≤0.5, a total of 79 differentially expressed flavonoid metabolites were found. With 24, 28, and 41 flavonoid metabolites identified in CK vs. GL, GL vs. YL, and CK vs. YL, respectively. Seven dems were detected in all three groups.

#### 3.2.1. Changes in Anthocyanins, Flavonols, Flavanones, and Isoflavones During Different Developmental Stages

Among anthocyanins, the contents of Cyanidin 3-*O*-glucoside (Kuromanin) and Malvidin in the YL group were 3.34 times and 6.63 times, respectively, that of the CK treatment. In contrast, the content of Cyanidin 3-*O*-galactoside in CK was extremely low during the sprouting period of maize, with almost undetectable signals. Relevant studies have shown that the anthocyanin synthesis stage is mainly divided into an early preparation stage and a late synthesis stage [40]. The early preparation stage involves the production of colorless anthocyanidins from colorless precursors, while the late synthesis stage involves the formation of various types of colored anthocyanidins and their derivatives from colorless anthocyanidins [41].

The contents of three flavonol differential metabolites showed a significant increasing trend (*p* < 0.05) before YL treatment. In the comparison between CK and YL, the contents of Kumatakenin and Aromadedrin (Dihydrokaempferol) in the YL group were 23.79 times and 24.83 times higher, respectively, than those in the CK group. Germination is a traditional, economical, and natural bioprocessing technique to enhance flavonoid content. For instance, mung bean sprouts exhibit higher total flavonoid levels than seeds [42]. Similarly, the content of polyphenols and flavonoids in lentil and lupin seeds also increases upon germination [43]. Wheat sprouts show a 2.25-fold increase in flavonoid content compared to pre-germination levels [44]. For mung bean, soybean, and black bean sprouts, total flavonoid content continuously rises during germination [45]. Quercetin 4′-*O*-glucoside (Spiraeoside) was abundant in CK but declined rapidly thereafter, becoming undetectable in YL and existing only during the early stages of maize development, playing a crucial role in plant antioxidant defense, as is consistent with related literature reports [46].

Among flavanones, 4′,5,7-Trihydroxyflavanone, Xanthohumol, Eriodictyol, and Naringenin were undetected in raw maize kernels but were present at higher levels during maize kernel germination. Research findings indicate that germinated waxy wheat boasts higher levels of dietary fiber, free amino acids, and total phenolic compounds than its ungerminated counterpart [47]. Germination also improves the texture of cooked brown rice, enhancing water absorption and softening the grains for better quality [5].

In isoflavones, Biochanin A and Sissotrin were undetected in raw maize kernels but were present at higher levels in YL, with Prunetin content in YL being 76.39 times higher than that in CK. Calycosin consistently showed a decreasing trend (Table 2).

#### 3.2.2. Changes in Flavonoid Components During Different Developmental Stages

A total of 36 flavonoid differential metabolites were identified in maize kernels from 3 different periods, exhibiting significant variations in their content trends. Specifically, eight metabolites including 4,2′,4′,6′-Tetrahydroxychalcone, *C*-hexosyl-apigenin *O*-*p*-coumaroylhexoside, Tricin 5-*O-β*-guaiacylglycerol, Tricin *O*-glycerylhexoside, Tricin *O*-sinapic acid, Eriodictyol *C*-hexoside, Butin, and Isovitexin 7-*O*-glucoside (saponarin) were undetectable in the raw maize kernels. However, they showed high concentrations during germination, which may be correlated with the continuous growth and development of the plant, where phenolic compounds are synthesized continuously, leading to a gradual increase in flavonoid content [48]. Experimental data indicates that antioxidant activity in germinated soybean seeds is 2.89 times higher than in unprocessed seeds [49]. Total phenolic content and antioxidant activity increase with the germination of quinoa, buckwheat, and wheat [50]. Peas and soybeans exhibit significantly increased antioxidant activity post-germination (*p* < 0.05), whereas lentils show a decline [51].

Among 16 metabolites, the concentrations of *C*-hexosyl-chrysoeriol *O*-hexoside, Tricin *O*-phenylformic acid, *C*-pentosyl-*C*-hexosyl-apigenin, Tricin 4′-*O*-(*β*-guaiacylglyceryl) ether 5-*O*-hexoside, Tricin 4′-*O*-syringyl alcohol, Tricin 7-*O*-hexoside, *C*-hexosyl-apigenin *C*-pentoside, Apigenin 6,8-*C*-diglucoside, 6,8-di-*C*-glucoside Apigenine, Isovitexin, 5,7-Dihydroxy-3′,4′,5′-trimethoxyflavone, Diosmin, Apiin, Vicenin-3, Schaftoside, and Isoschaftoside decreased significantly at the GL stage but increased significantly after GL (*p* < 0.05).

As the germination process progressed, *C*-hexosyl-luteolin *O*-hexoside, Acacetin *O*-acetyl hexoside, Tricin 5-*O*-hexoside, Luteolin 8-*C*-hexosyl-*O*-hexoside, di-*C,C*-hexosyl-apigenin, Apigenin 6-*C*-pentoside, Puararin, and Persicogenin were present at higher levels in the CK stage but subsequently disappeared. Whereas 8-*C*-hexosyl-luteolin *O*-pentoside, Acacetin, Tricin *O*-eudesmic acid, and Limocitrin *O*-hexoside showed a consistent upward trend in their concentrations (Table 3).

### 3.3. KEGG Pathway Enrichment Analysis for Metabolites

**Figure 3:**
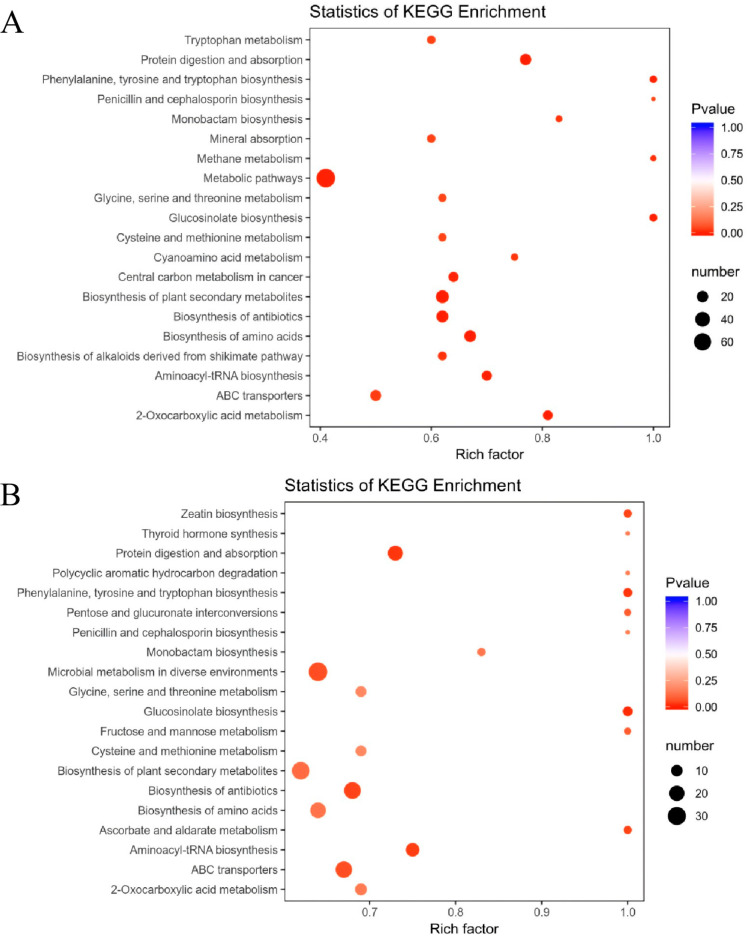

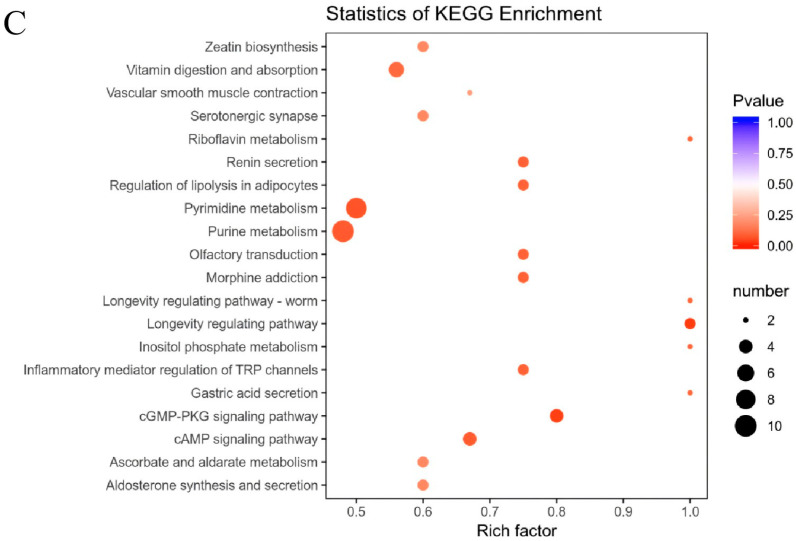
KEGG pathway analysis for CK vs. GL (**A**), CK vs. YL (**B**), and GL vs. YL (**C**).

Through the pathway enrichment analysis of differential metabolites, it was revealed that the comparison of CK vs. GL is primarily enriched in metabolic pathways, the biosynthesis of plant secondary metabolites (Figure 3A), and the biosynthesis of antibiotics. The comparison of CK vs. YL is mainly enriched in microbial metabolism in diverse environments, the biosynthesis of plant secondary metabolites (Figure 3B), and the biosynthesis of antibiotics. Meanwhile, the comparison of GL vs. YL is primarily enriched in pyrimidine metabolism and purine metabolism pathways (Figure 3C).

### 3.4. Transcriptome Analysis

RNA-Seq generated 52,303,489, 50,603,542, and 46,922,697 clean reads from the JP (juvenile plant), GC (germinating corn), and YC (young corn) libraries, respectively. The total base count exceeds 6 GB, with a Q30 percentage (sequences with a sequencing error rate below 0.1%) greater than 91%. The average GC content is 57%. Overall, the data indicates high sequencing quality, suitable for further analysis.

**Figure 4:**
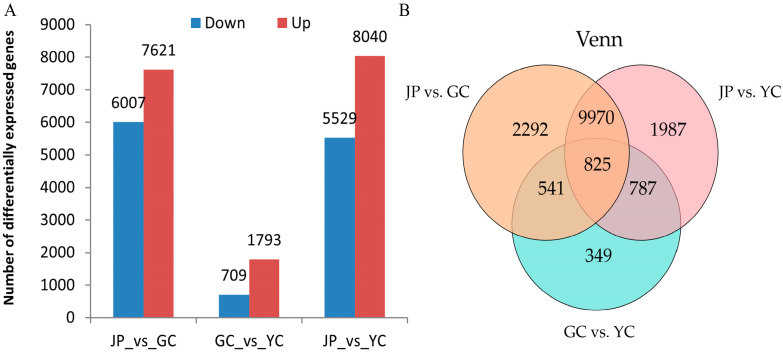
Analysis of number of DEGs in JP, GC, and YC (**A**). Venn diagram analysis of DEGs in JP, GC, and YC (**B**).

After filtering, 13,628, 2502, and 13,569 differentially expressed genes (DEGs) were identified between JP vs. GC, GC vs. YC, and JP vs. YC, respectively. When comparing 2 different stages, 7621, 1793, and 8040 genes were upregulated, and 6007, 709, and 5529 genes were downregulated in JP relative to GC, GC relative to YC, and JP relative to YC, respectively (Figure 4A). These data suggest that the majority of DEGs in maize kernels are upregulated during the sprouting period. A Venn diagram analysis reveals a shared 825 DEGs among the 3 groups (Figure 4B).

### 3.5. Integrated Analysis

#### 3.5.1. KEGG Metabolic Pathway Analysis

**Figure A2:**
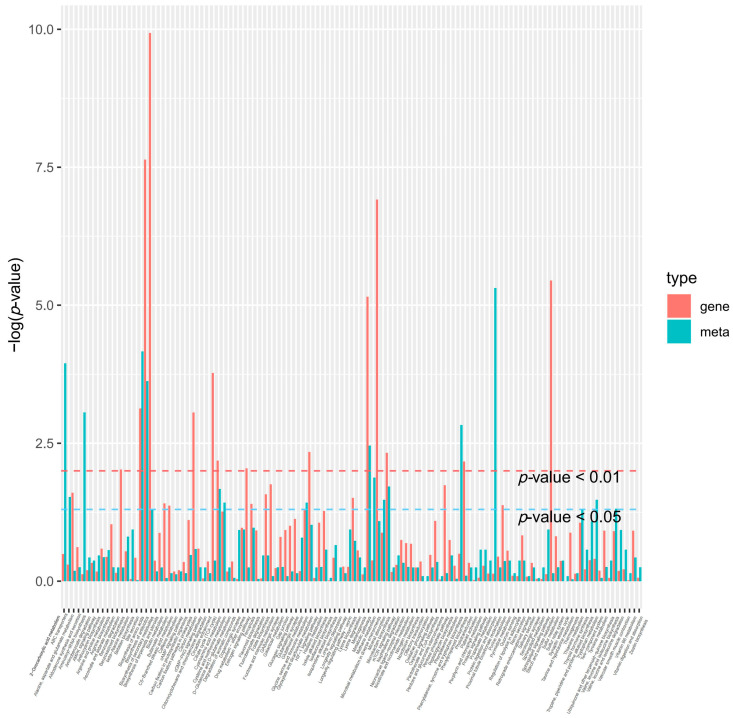
KEGG analysis of control group vs. germinating stage.

A bar chart was constructed based on the enrichment levels of differential metabolites and differential gene pathways. For the comparison between the control group and the germinating stage, DEGs were mapped to 306 KEGG pathways (Appendix A Figure A2). Among them, the largest number of DEGs were mapped to metabolic pathways (ko01100, with 1084 genes).

**Figure A3:**
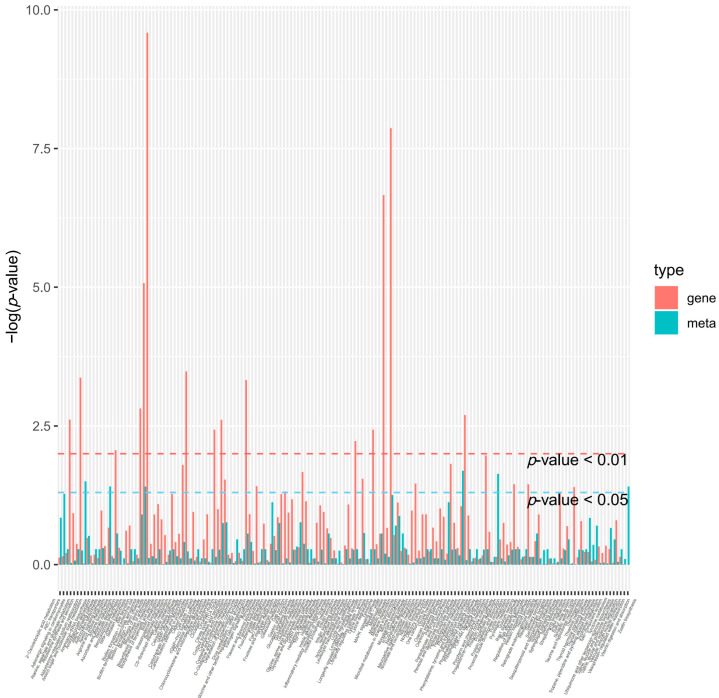
KEGG analysis of control group vs. young corn stage.

By comparing the DEGs between the control group and the young corn stage, 306 KEGG pathways were identified (Appendix A Figure A3). Here, the highest number of DEGs were mapped to metabolic pathways (ko01100, with 1087 genes), followed by the biosynthesis of secondary metabolites (ko01110, 669 genes), the biosynthesis of antibiotics (ko01130, 263 genes), carbon metabolism (ko01200, 144 genes), and amino sugar and nucleotide sugar metabolism (ko00520, 87 genes).

**Figure A4:**
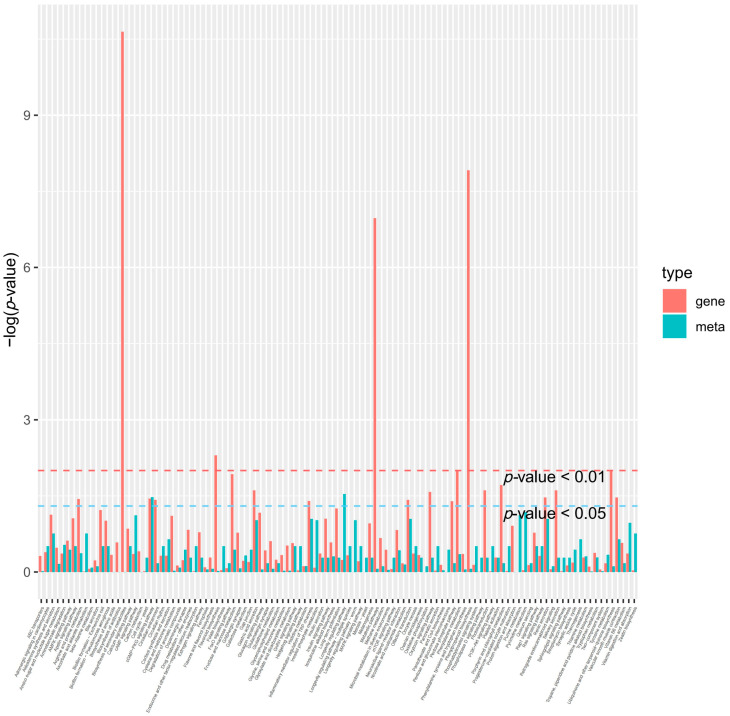
KEGG analysis of germinating stage vs. young corn stage.

Comparing the DEGs between the germinating stage and the young corn stage, 237 KEGG pathways were identified (Appendix A Figure A4). The most significant enrichment was observed in metabolic pathways (ko01100, with 238 genes), followed by the biosynthesis of secondary metabolites (ko01110, 166 genes), phenylpropanoid biosynthesis (ko00940, 47 genes), photosynthesis (ko00195, 25 genes), and stilbenoid, diarylheptanoid, and gingerol biosynthesis (ko00945, 16 genes). Additionally, some genes participate in various metabolic pathways, each playing a distinct role in regulating the synthesis of phenolic compounds [52]. All flavonoids are derived from the shikimic acid pathway, a common pathway that leads to the production of aromatic amino acids such as phenylalanine [53].

#### 3.5.2. Combined Analysis of Flavonoids

Flavonoids are significant metabolites in corn kernels, with germinated kernels boosting free, bound phenolic acids, and bound flavonoid content by 169%, 230%, and 311%, respectively [10]. To date, a total of 53 differential metabolites of polyphenolic compounds were identified across the various groups, with 11 of these differential metabolites remaining unannotated to specific metabolic pathways. The remaining 42 phenolic compounds are primarily annotated to 4 metabolic pathways, and 16 flavonoid metabolites are similarly mapped to these 4 KEGG pathways, involving 40 related genes. The differential metabolites include six flavonoids: Diosmin, Butin, Apiin, 4,2′,4′,6′-Tetrahydroxychalcone, Apigenin 6-*C*-pentoside, and Isoschaftoside; four isoflavones: Calycosin, Prunetin, Biochanin A, and Sissotrin; three flavanones: Eriodictyol, Homoeriodictyol, and Naringenin; two flavonols: Dihydroquercetin and Aromadedrin (Dihydrokaempferol); and one Anthocyanin: Cyanidin 3-*O*-glucoside (Kuromanin). The four distinct KEGG pathways are ko00940 (phenylpropanoid biosynthesis), ko00941 (flavonoid biosynthesis), ko00942 (anthocyanin biosynthesis), and ko00943 (Isoflavonoid biosynthesis).

**Figure 5:**
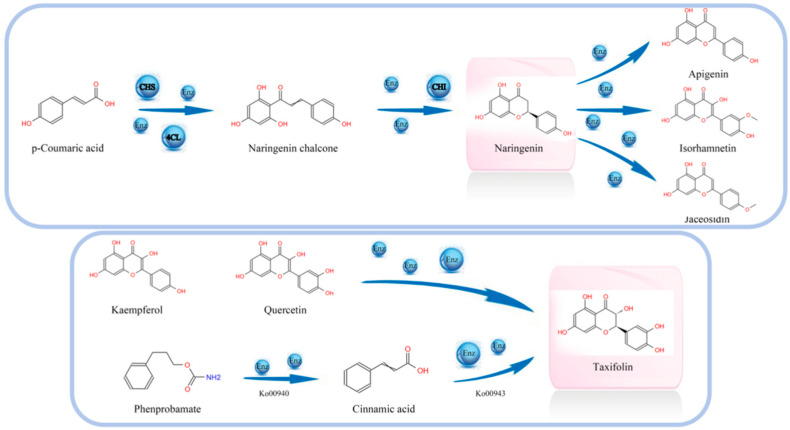
Analysis of metabolite transformation pathway.

No association among genes was detected for acacetin, viochanin A, calycosin, homoeriodictyol, isovitexin, prunetin, and xanthohumol. We only found that one gene had an association with Apiin, Cyanidin 3-*O*-glucoside (Kuromanin) or Sissotrin. Moreover, Eriodictyol, aromadedrin (Dihydrokaempferol), and 4,2′,4′,6′-tetrahydro-xychalcone were found to be regulated by three, four, or five genes, respectively. Dihydroquercetin (Taxifolin) is the raw material of upscale food, a powerful antioxidant, and is recognized as an important and effective vitamin P, which could promote the improvement of skin protection and avoid and reduce internal and external toxins, radiation, microbes, viruses, and other damage, which can come from three different substances and can change into three different metabolites; we found that six genes can regulate it in the study. We also found that eight genes had influence on butin; however, butin can only change into other one metabolite and may come from other two different compounds. Naringenin has antibacterial, anti-inflammatory, free radical scavenging, antioxidant, and other functions, and can be widely used in medicine, food, and other fields; we found that 10 genes can regulate it in this study, and naringenin only came from 1 substance, but can be changed into 7 different metabolites (Figure 5).

## 4. Conclusions

In this study, the germination process of corn kernels has resulted in the production of numerous flavonoids that were absent initially, demonstrating the significant benefits of germination in enhancing the nutritional function of corn kernels. Specifically, 16 flavonoids were identified and primarily annotated to 4 metabolic pathways, involving 40 related genes. Among the differential metabolites, Dihydroquercetin (Taxifolin) is regulated by six distinct differential genes, while Naringenin is regulated by ten differential genes, leading to the transformation into seven different metabolites.

## Figures and Tables

**Table 1:** The numbers of different kinds of flavonoid metabolites.

Component Name	Number
Anthocyanins	3
Flavone	61
Flavonol	12
Flavanone	13
Isoflavone	6
Sum	95

**Table 2:** Differentially accumulated anthocyanins, flavanone, flavonol, and isoflavone compounds in maize kernels of different development periods.

Serial Number	Component/ Metabolite Name	Content			Fold Change			VIP		
		CK	GL	YL	GL/CK	YL/GL	YL/CK	CK vs. GL	GL vs. YL	CK vs. YL
	Anthocyanin									
1	Malvidin	7.11 × 10^3^	3.85 × 10^4^	4.72 × 10^4^	5.41	-	6.63	1.298	-	1.092
2	Cyanidin 3-*O*-glucoside (Kuromanin)	1.44 × 10^4^	3.47 × 10^4^	4.80 × 10^4^	2.41	-	3.34	1.363	-	1.151
3	Cyanidin 3-*O*-galactoside	3.35 × 10^5^	1.27 × 10^5^	-	0.38	0	0	1.325	1.325	1.179
	Flavonol									
1	Di-*O*-methylquercetin	6.94 × 10^6^	-	2.43 × 10^6^	-	-	0.35	-	-	1.139
2	Aromadedrin (Dihydrokaempferol)	2.83 × 10^4^	4.04 × 10^4^	7.03 × 10^5^	-	17.41	24.83	-	1.067	1.009
3	Kumatakenin	3.40 × 10^4^	4.74 × 10^4^	8.09 × 10^5^	-	17.08	23.79	-	1.276	1.155
4	Quercetin 4′-*O*-glucoside (Spiraeoside)	7.30 × 10^5^	-	-	-	-	0	-	-	1.177
5	Dihydroquercetin (Taxifolin)	-	1.15 × 10^4^	2.10 × 10^5^	-	18.28	-	-	-	1.064
	Flavanone									
1	4′,5,7-Trihydroxyflavanone	-	3.62 × 10^4^	9.90 × 10^4^	4022.22	-	11003.7	1.39	-	1.175
2	Homoeriodictyol	1.20 × 10^5^	-	5.00 × 10^4^	-	-	0.42	-	-	1.029
3	Xanthohumol	-	3.10 × 10^4^	4.59 × 10^4^	3444.44	-	5096.3	1.399	-	1.179
4	Eriodictyol	-	-	2.06 × 10^5^	-	-	22,888.89	-	-	1.178
5	Naringenin	-	3.03 × 10^4^	7.23 × 10^4^	3370.37	-	8029.63	1.392	-	1.174
	Isoflavone									
1	Prunetin	7.09 × 10^3^	1.62 × 10^4^	5.41 × 10^5^	2.28	33.48	76.39	1.235	1.259	1.144
2	Calycosin	3.59 × 10^4^	3.12 × 10^4^	8.01 × 10^3^	-	0.26	0.22	-	1.286	1.154
3	Biochanin A	-	-	1.66 × 10^5^	-	18,485.19	18,485.19	-	1.319	1.173
4	Sissotrin	-	3.84 × 10^3^	8.02 × 10^4^	-	20.9	8914.81	-	1.038	1.167

Note: A metabolite fold change value > 1.0 indicates an increase in fold, while a value < 1.0 indicates a decrease. Differentially accumulated phenolic compounds were identified using the thresholds of VIP ≥ 1 and |log_2_ fold change| ≥ 2 (upregulation) or ≤0.5 (downregulation).

**Table 3:** Differences in flavonoid accumulation in maize kernels during different developmental stages.

Serial Number	Component/ Metabolite Name	Content			Fold Change			VIP		
		CK	GL	YL	GL/CK	YL/GL	YL/CK	CK vs. GL	GL vs. YL	CK vs. YL
1	4,2′,4′,6′-Tetrahydroxychalcone	-	4.92 × 10^4^	1.24 × 10^5^	5462.96	-	13,777.78	1.392	-	1.174
2	8-*C*-hexosyl-luteolin *O*-pentoside	5.70 × 10^4^	1.35 × 10^5^	5.33 × 10^5^	2.38	3.94	9.36	1.265	1.206	1.146
3	*C*-hexosyl-chrysoeriol *O*-hexoside	3.99 × 10^5^	2.48 × 10^5^	9.42 × 10^5^	-	3.79	2.36	-	1.268	1.039
4	*C*-hexosyl-luteolin *O*-hexoside	4.18 × 10^4^	1.88 × 10^4^	-	0.45	-	-	1.146	-	-
5	*C*-hexosyl-apigenin *O*-p-coumaroylhexoside	-	1.40 × 10^4^	5.73 × 10^4^	-	4.1	-	-	1.285	-
6	*C*-pentosyl-*C*-hexosyl-apigenin	2.95 × 10^5^	-	7.02 × 10^5^	-	-	2.38	-	-	1.03
7	Tricin *O*-phenylformic acid	7.41 × 10^3^	-	3.82 × 10^4^	-	-	5.15	-	-	1.132
8	Acacetin	9.87 × 10^3^	1.91 × 10^4^	6.36 × 10^5^	-	33.3	64.42	-	1.255	1.139
9	Acacetin *O*-acetyl hexoside	1.32 × 10^5^	1.04 × 10^5^	4.36 × 10^4^	-	0.42	0.33	-	1.31	1.148
10	Tricin 4′-*O*-(*β*-guaiacylglyceryl) ether 5-*O*-hexoside	6.04 × 10^4^	-	-	-	-	0	-	-	1.178
11	Tricin 4′-*O*-syringyl alcohol	1.29 × 10^4^	-	5.99 × 10^4^	-	-	4.66	-	-	1.136
12	Tricin 5-*O*-hexoside	2.20 × 10^5^	5.72 × 10^4^	3.43 × 10^4^	0.26	-	0.16	1.274	-	1.16
13	Tricin 5-*O-β*-guaiacylglycerol	-	7.43 × 10^4^	2.49 × 10^5^	-	3.35	-	-	1.096	-
14	Tricin 7-*O*-hexoside	1.58 × 10^5^	4.99 × 10^4^	4.00 × 10^5^	0.32	8.02		1.243	1.281	
15	Tricin *O*-glycerylhexoside	-	-	2.87 × 10^5^	-	-	31,888.89	-	-	1.179
16	Tricin *O*-sinapic acid	-	-	3.06 × 10^4^	-	3396.3	3396.3	-	1.326	1.179
17	Tricin *O*-eudesmic acid	4.64 × 10^3^	5.89 × 10^3^	1.68 × 10^4^	-	2.85	3.61	-	1.074	1.126
18	Limocitrin *O*-hexoside	1.29 × 10^4^	9.15 × 10^4^	2.51 × 10^5^	7.11	2.74	-	1.07	1.202	
19	Luteolin 8-*C*-hexosyl-*O*-hexoside	1.95 × 10^5^	3.12 × 10^4^	-	0.16	-	-	1.35	-	-
20	Apigenin 6-*C*-pentoside	3.05 × 10^4^	-	-	-	-	0	-	-	1.176
21	di-*C,C*-hexosyl-apigenin	2.89 × 10^4^	-	-	0	-	-	1.399	-	-
22	*C*-hexosyl-apigenin *C*-pentoside	2.62 × 10^5^	1.06 × 10^5^	5.91 × 10^5^	0.4	5.59	2.26	1.061	1.247	1.013
23	Apigenin 6,8-*C*-diglucoside	3.29 × 10^5^	1.46 × 10^5^	6.86 × 10^5^	0.44	4.7	2.09	1.082	1.252	1.014
24	6,8-di-*C*-glucoside Apigenine	1.07 × 10^6^	4.73 × 10^5^	2.80 × 10^6^	0.44	5.93	2.62	1.124	1.283	1.06
25	Eriodictyol *C*-hexoside	-	-	3.18 × 10^4^	-	3533.33	3533.33	-	1.325	1.178
26	Isovitexin	1.87 × 10^4^	-	4.70 × 10^4^	-	-	2.51	-	-	1.01
27	Butin	-	4.31 × 10^4^	1.24 × 10^5^	4788.89	-	13,807.41	1.389	-	1.174
28	5,7-Dihydroxy-3′,4′,5′-trimethoxyflavone	8.46 × 10^3^	-	1.75 × 10^4^	-	-	2.06	-	-	1.002
29	Diosmin	4.34 × 10^4^	-	1.07 × 10^5^	-	-	2.47	-		1.024
30	Puararin	2.45 × 10^4^	1.44 × 10^4^	-	-	0	0	-	1.324	1.179
31	Apiin	3.39 × 10^5^	3.17 × 10^5^	7.46 × 10^5^	-	2.35	2.2	-	1.105	1.065
32	Persicogenin	2.45 × 10^4^	9.88 × 10^3^	-	0.4	-	-	1.145	-	-
33	Vicenin-3	3.36 × 10^5^	1.47 × 10^5^	4.97 × 10^5^	0.44	3.38	-	1.008	1.194	-
34	Schaftoside	4.36 × 10^5^	1.77 × 10^5^	6.41 × 10^5^	0.41	3.63	-	1.131	1.235	-
35	Isoschaftoside	3.28 × 10^5^	1.32 × 10^5^	5.10 × 10^5^	0.4	3.86	-	1.117	1.247	-
36	Isovitexin 7-*O*-glucoside (Saponarin)	-	1.66 × 10^5^	5.83 × 10^5^	18,403.7	3.52	64,740.74	1.397	1.184	1.179

## Data Availability

The original contributions presented in the study are included in the article; further inquiries can be directed to the corresponding author.

## Abbreviations

CK, raw maize kernel; GL, germination period; YL, sprouting period; JP, represents juvenile plant; GC, represents germinating stage corn; YC, represents young corn.
